# Supplementary figures and images for: Heat Stress Impact on Yield and Composition of Quinoa Straw under Mediterranean Field Conditions
Source: Plants (Basel). 2021 May 11;10(5):955. doi: 10.3390/plants10050955 (PMC8150704; doi:10.3390/plants10050955)

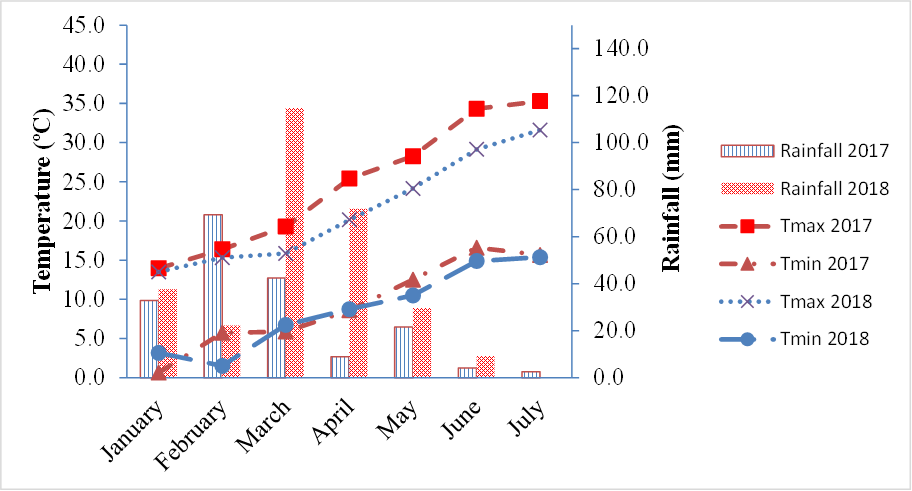

Supplement: Supplementary file 1 [file plants-10-00955-s001.zip › plants-1197610-supplementary.tif]
